# Tumor Biology, Genetic Risk, and Systemic Therapy in Familial Breast Cancer: An 8-Year Experience from a German Center for Familial Breast and Ovarian Cancer

**DOI:** 10.1159/brc/abrag005

**Published:** 2026-08-25

**Authors:** Maria Eleni Hatzipanagiotou, Lisa Witterauf, Mikosch Augele, Aviva Fischer, Ivana Holzhauser, Ines Schönbuchner, Simone Hammer, Miriam Fernandez-Pacheco, Deborah Huber, Bernhard H.F. Weber, Davor Lessel, Olaf Ortmann, Stephan Seitz

**Affiliations:** a Department of Gynecology and Obstetrics, Comprehensive Cancer Center (CCC) Würzburg, Erlangen, Regensburg, Augsburg (WERA), National Center for Tumor Diseases (NCT), University Medical Center Regensburg, Regensburg, Germany; b Department of Radiology, University Hospital Regensburg, Regensburg, Germany; c Department of Obstetrics and Gynecology, General Hospital Neumarkt, Akademisches Lehrkrankenhaus der Friedrich-Alexander-Universität Erlangen-Nürnberg, Erlangen, Germany; d Institute of Human Genetics, University of Regensburg, Regensburg, Germany; e Institute of Clinical Human Genetics, University Hospital Regensburg, Regensburg, Germany; f Hospital Dritter Orden, Obstetrics and Gynaecology, Munich, Germany

**Keywords:** Hereditary breast cancer, *BRCA1/2* mutations, Triple-negative breast cancer, Neoadjuvant chemotherapy, Pathological complete response, Platinum-based therapy

## Abstract

**Introduction:**

Approximately 30% of breast cancer cases involve a significantly increased familial risk, often associated with pathogenic germline mutations. This study evaluates the spectrum of pathogenic variants identified, systemic therapy patterns, tumor biology, and treatment response among breast cancer patients treated at the Regensburg Center of the German Consortium for Hereditary Breast and Ovarian Cancer (GC-HBOC) between 2012 and 2020.

**Methods:**

A retrospective analysis was performed on 385 breast cancer patients who met the GC-HBOC criteria for elevated familial risk. Patients were categorized based on genetic status (pathogenic *BRCA1/2-*variants, pathogenic variants in other breast and ovarian cancer-related genes such as *CHEK2*, *ATM*, *PALB2*, *RAD51C*, *RAD51D*, or *BARD1* [*BRCA*-related], or negative) and tumor subtype (triple-negative breast cancer [TNBC], hormone receptor-positive/HER2-negative [HR+/HER2−], or HER2-positive [HER2+]). The use of chemotherapy, including platinum-based regimens, and rates of pathological complete response (pCR) following neoadjuvant chemotherapy (NACT) were analyzed.

**Results:**

In this study, pathogenic variants in established breast and ovarian cancer-predisposing genes were identified in 49%, primarily in *BRCA1* and *BRCA2* (36%). TNBC was predominant in *BRCA1*-positive patients (61%), whereas *BRCA2*-positive and variant-negative patients more commonly presented with HR+/HER2− tumors. Chemotherapy was administered in 76% of patients, with the highest rate in *BRCA1* carriers (89%). The use of platinum-based chemotherapy increased significantly among TNBC patients after 2014, reaching 100% by 2020. Among 144 patients who received NACT, 22% achieved a pCR. *BRCA1*-positive patients had the highest pCR rate (35%), with TNBC/*BRCA1*-positive patients achieving a pCR in 67% of cases.

**Conclusion:**

The study demonstrates the effective implementation of genetic testing and guideline-based therapy in a real-world, high-risk breast cancer cohort. *BRCA1*-associated TNBC exhibited the highest chemotherapy sensitivity, as evidenced by elevated pCR rates. However, incomplete documentation of treatment outcomes somewhat limits the strength of the latter. Overall, these findings reinforce the value of personalized treatment strategies in hereditary breast cancer and underscore the need for more comprehensive real-world data collection to optimize patient management.

## Introduction

Breast cancer is the most common malignancy among women worldwide. In Germany, approximately 70,000 women and 700 men are diagnosed with breast cancer annually [1]. Around 30% of cases are associated with a significantly elevated familial risk compared with the general population [2]. Among individuals with a family history of breast or ovarian cancer, pathogenic *BRCA1* or *BRCA2* variants are detectable in roughly 25%, while an additional 5% carry pathogenic variants in moderate-risk genes such as *CHEK2*, *ATM*, *PALB2*, *RAD51C*, *RAD51D*, or *BARD1* [3, 4]. These germline mutations serve as important biomarkers for targeted therapy [5], prevention [6], and risk-reducing surgery [7].

Given the typically earlier onset of disease and limitations of conventional screening, women with markedly increased familial risk are offered intensified surveillance protocols, including breast MRI, as standard screening methods may be insufficient [6]. Since 1995, the German Consortium for Hereditary Breast and Ovarian Cancer (GC-HBOC) has provided interdisciplinary care to over 40,000 families with hereditary cancer predisposition through 23 university-based centers across Germany. The consortium follows standardized protocols for risk assessment, genetic counselling and testing, surveillance, and prevention strategies [3]. To support comprehensive genetic evaluation, the GC-HBOC developed the TruRisk^®^ gene panel, which is continuously updated to reflect the latest scientific knowledge [4].

The Regensburg Center for Familial Breast and Ovarian Cancer (FBREK), a member of the GC-HBOC, has offered a comprehensive program for high-risk individuals since 2011. Services include medical counseling, molecular genetic diagnostics, enrollment in intensified surveillance programs, and guidance on risk-reducing surgery and systemic therapy. The center currently provides care to over 700 patients. In 2020, the Regensburg FBREK Center became one of the first in the network to be certified by OnkoZert^®^ GmbH, the certification body of the German Cancer Society, in recognition of its adherence to evidence-based care standards.

Despite the availability of standardized guidelines, real-world data remain limited on how patients at high risk for developing breast cancer who subsequently develop the disease are managed regarding genetic testing and systemic therapy, particularly chemotherapy (CT) regimens. Moreover, the influence of genetic status on treatment patterns and outcomes, especially in relation to platinum-based therapies and pathological complete response (pCR), is not well characterized.

This study aims to systematically evaluate the management of high-risk breast cancer patients treated at the Regensburg Center, with a focus on germline genetic testing and systemic CT. Specific objectives include (1) analyzing the distribution of tumor biology subtypes according to genetic risk status, (2) assessing temporal trends in systemic therapy, with emphasis on platinum-based regimens, and (3) evaluating treatment response, as measured by pCR rates, across genetic and biological subgroups.

## Methods

### Study Population and Variables

This retrospective cohort study was based on patient data obtained from multiple complementary sources. The majority of the data were derived from the registry documentation system of the German Consortium for Hereditary Breast and Ovarian Cancer (GC-HBOC; BRCA-2006 registry), particularly with regard to genetic findings and familial risk information. In addition, data were extracted from the hospital and patient documentation system, Medical Control Center (MCC; Meierhofer AG), at our center. To ensure completeness of the dataset, additional information was retrieved from the patient management software Medical Office (INDAMED) as well as from patient records at the Institute of Human Genetics, University Hospital Regensburg. Thus, the GC-HBOC registry did not serve as the sole data source but was integrated with institutional clinical documentation systems and patient records. Standardized registry variables were incorporated where available; however, the final dataset was constructed based on predefined study variables combining information from all sources. Patients were not exclusively prospectively registered within the GC-HBOC database. While many patients were enrolled as part of routine clinical care, the registry alone did not provide all variables required for this study. Therefore, additional institutional documentation systems and patient records were used to complete the dataset.

The study cohort included both patients treated at our institution and patients who were referred to the Regensburg FBREK Center for genetic counseling and testing while receiving oncologic treatment at external institutions. For patients treated externally, clinical and treatment-related data were available only to the extent documented at the time of presentation to our center. The dataset was analyzed to assess genetic testing, tumor biology, and treatment characteristics among high-risk breast cancer patients. Inclusion criteria for familial risk followed the GC-HBOC guidelines [4]. Data were collected from female patients with breast cancer who were treated or counseled between January 1, 2012, and December 30, 2020, and who met the criteria for elevated familial risk for breast and ovarian cancer. In total, 467 patient records were reviewed. Exclusions were made for patients diagnosed with ovarian cancer only (*n* = 73), benign tumors (*n* = 2), or carcinoma in situ (*n* = 7), resulting in a final study cohort of 385 breast cancer patients.

### Genetic Testing and Evolution of Panel Diagnostics

Genetic testing at our center evolved substantially during the study period in parallel with advances in hereditary cancer diagnostics. Until 2015, genetic testing was limited to *BRCA1* and *BRCA2.* Starting in 2015, multigene panel testing was introduced and subsequently expanded. Between 2015 and 2017, the panel included *BRCA1*, *BRCA2*, *CHEK2*, *ATM*, *PALB2*, *RAD51C*, *RAD51D*, *CDH1*, *NBN*, and *TP53*. From January 2018 onward, the panel was modified by replacing *NBN* with *BRIP1*, while all other genes remained unchanged. Since 2019, the panel has been further expanded to the TruRisk^®^ gene panel. The panel analyzes all currently established risk genes for hereditary breast, ovarian, and endometrial cancer (e.g., *ATM*, *BRCA1*, *BRCA2*, *BRIP1*, *CDH1*, *CHEK2*, *EPCAM*, *MLH1*, *MSH2*, *MSH6*, *PALB2*, *PMS2*, *PTEN*, *RAD51C*, *RAD51D*, *STK11*, *TP53*), as well as genes requiring further research (e.g., *BARD1*, *NBN*, *FANCM*, *XRCC2*, *RECQL*) [4]. Thus, over time, genetic testing progressed from *BRCA1/2*-only analysis to a comprehensive multigene panel approach, as implemented within the GC-HBOC framework [4]. Patients who had undergone genetic testing prior to 2015 were invited to undergo expanded multigene panel testing through a structured recall system. The uptake of retesting was not analyzed within the scope of the present study but has been investigated in a separate project conducted at the same center.

Genetic testing was performed according to the GC-HBOC inclusion criteria valid at the time of presentation. These criteria have changed only slightly since 2012, with minor expansions such as the extension of the age threshold for genetic testing in triple-negative breast cancer (TNBC) from 50 to 60 years and more recently to 70 years. Patients who did not meet the inclusion criteria for genetic testing at the time of their diagnosis, but who would meet current criteria, were not systematically identified or re-invited for genetic evaluation at our center.

### Statistical Analysis

Data analysis was conducted using anonymized and aggregated patient data recorded in Microsoft Excel 2013 [8]. Patients were categorized according to genetic mutation status and tumor biology. Based on the results of genetic testing, the cohort was stratified into 3 main groups: (1) patients with a pathogenic *BRCA1* or *BRCA2* mutation (“*BRCA1/2* positive”); (2) patients with no detected pathogenic mutation (“negative”); and (3) patients with other *BRCA*-related pathogenic mutations (“other mutations”). Within the *BRCA1/2*-positive group, a further subdivision distinguished between *BRCA1*-positive and *BRCA2*-positive patients to allow more specific analyses. Two additional datasets (“*BRCA1* positive” and “*BRCA2* positive”) were created for this purpose. Patients harboring pathogenic variants in both *BRCA1* and *BRCA2* were included in both datasets. These datasets were further subdivided according to tumor biology, resulting in 4 additional tables for each of the genetic subgroups: triple-negative breast cancer (“TNBC”), lacking hormone receptor expression and HER2 amplification; hormone receptor-positive, HER2-negative tumors (“HR+, HER2−”); HER2-positive tumors (“HER2+”), and patients for whom a clear tumor subtype could not be determined due to missing histopathological data (“not classifiable”).

The complete dataset was duplicated to first categorize tumors by subtype, and subsequently by the presence of specific genetic mutations. All datasets were structured uniformly and included the following information: result of genetic testing, date of initial diagnosis, and histopathological findings from both core needle and postoperative biopsies, including progesterone receptor expression, estrogen receptor expression, and HER2 receptor status. Tumor and lymph node status were recorded according to the TNM classification. Information on CT was also collected, specifying the type of administration (neoadjuvant or adjuvant) and whether platinum-based agents were used (yes, no, or unknown). Figure 1 illustrates the study cohort and its respective subgroups. In addition to descriptive analyses, exploratory inferential statistical analyses were performed. Associations between categorical variables, including tumor subtype distribution and pCR rates across genetic subgroups, were analyzed using chi-square tests or Fisher’s exact tests where appropriate. Due to the retrospective design, missing follow-up data, and limited subgroup sizes, all inferential analyses were considered exploratory and hypothesis-generating. A two-sided *p* value <0.05 was considered statistically significant.

**Fig. 1.**
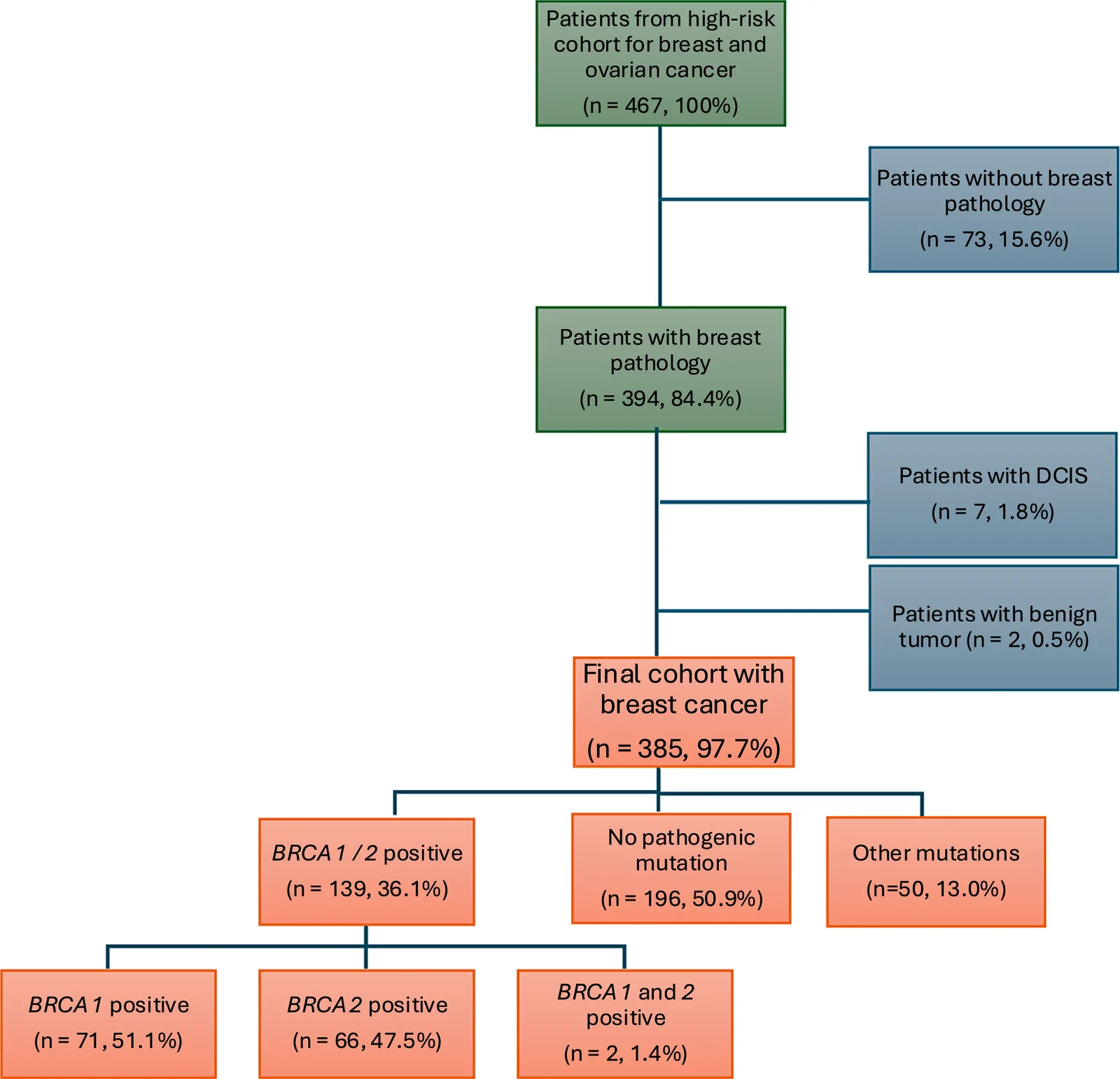
Overview of the study cohort.

## Results

### Distribution of Pathogenic *BRCA* Variants

A total of 385 breast cancer patients who received treatment or genetic counseling at the Center in Regensburg between January 1, 2012, and December 30, 2020, were included in the analysis. Table 1 summarizes the baseline characteristics of the cohort. Pathogenic *BRCA1* and/or *BRCA2* variants were detected in 36% of patients (139/385), while an additional 13% (50/385) carried pathogenic variants in other BRCA genes. Approximately half of the tested patients (196/385) had negative genetic test results. Among patients with pathogenic *BRCA1/2* variants, 51% (71/139) were *BRCA1* positive, 47% (66/139) were *BRCA2* positive, and 1% (2/139) were harbored pathogenic variants in both genes. Figure 2 provides a detailed breakdown of the “other” *BRCA* group, which comprised 50 patients with less common pathogenic variants. The most frequently altered genes were *CHEK2* (34%, 17/50), *PALB2* (22%, 11/50), and *ATM* (20%, 10/50). Single cases harbored pathogenic variants in *RAD51C*, *BARD1*, *BRIP1*, *FANCM*, *NBN*, and *CDH1*, while pathogenic variants in *TP53* were identified in 2 patients. Additionally, 2 patients were found to harbor two pathogenic variants in *ATM* and *BARD1*, respectively.

**Table 1.** Baseline characteristics of the cohort

Characteristic	Total cohort (*n* = 385)
Age at diagnosis	
Mean age, years	40.1
Median age, years	39
Range, years	16–69
Genetic testing results (%)	
BRCA1/2 pathogenic variant	139 (36)
Other pathogenic variants	50 (13)
No pathogenic mutation detected	196 (51)
Tumor biology (%)	
TNBC	100 (30)
HER2-positive	78 (23)
HR+/HER2−	160 (47)
Postoperative tumor stage (%)	
pTis/ypTis	11 (4)
pT1/ypT1	125 (52)
pT2/ypT2	72 (30)
pT3/ypT3	10 (4)
Missing/unavailable	23 (10)
Postoperative nodal stage (%)	
pN0/ypN0	159 (66)
pN1/ypN1	56 (23)
pN2/ypN2	17 (7)
pN3	6 (3)
Missing/unavailable	3 (1)
Systemic therapy (%)	
Any CT	257/338 (76)
NACT	144/257 (56)
Adjuvant CT	105/257 (41)
Unknown treatment setting	8/257 (3)

**Fig. 2.**
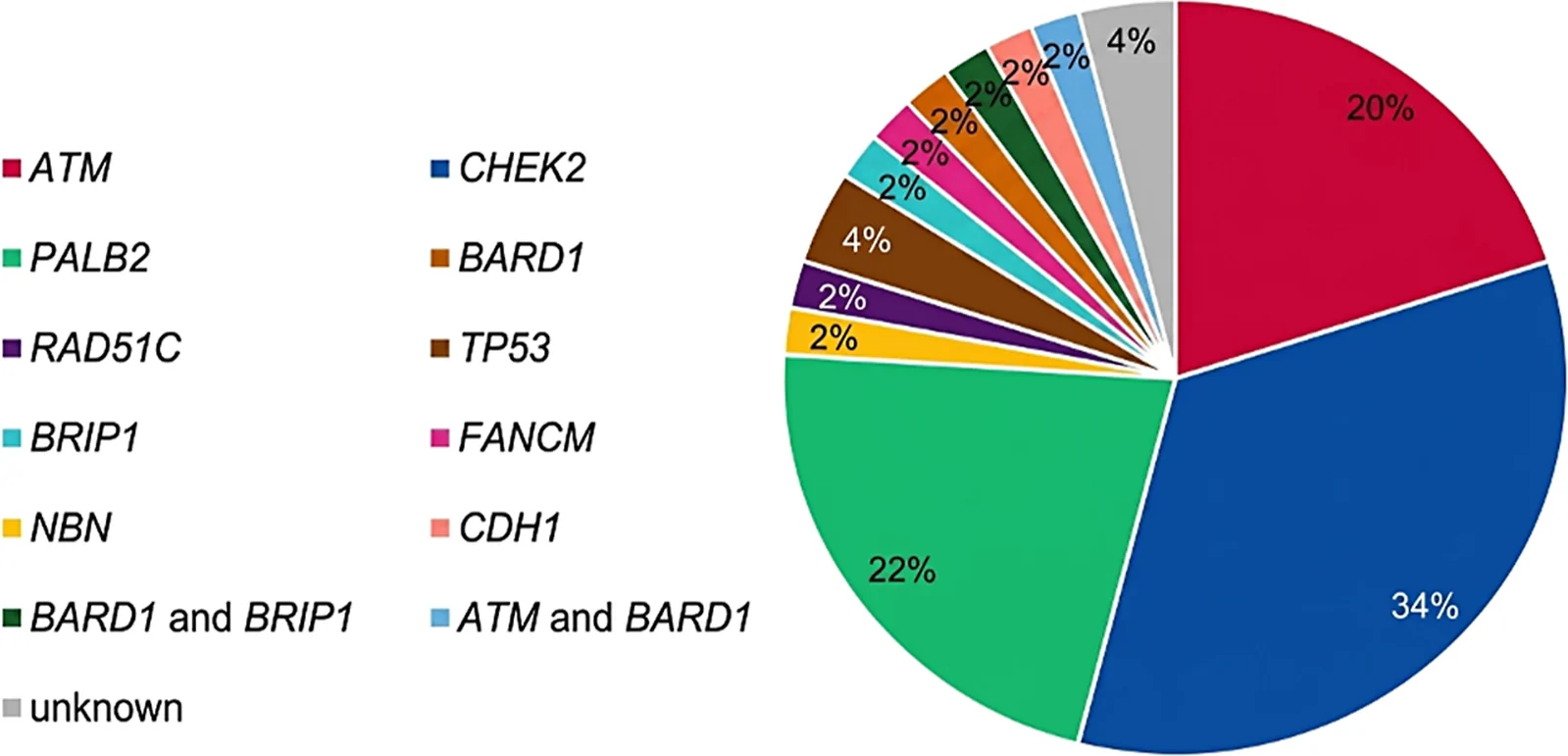
Non-BRCA1/BRCA2 pathogenic variants detected in the study cohort (*n* = 50).

### Tumor Biology

The cohort was stratified into 3 subgroups based on tumor biology. In 2 patients, multiple tumor foci with differing tumor biology were identified in the breast, and these tumors were therefore assigned to separate subgroups. Overall, 338 individual carcinomas were analyzed. Patients with HR+/HER2− carcinomas constituted the largest subgroup, representing 47% (160/338) of all tumors. TNBC accounted for 30% (100/338), while HER2-positive carcinomas were observed in 23% (78/338) of cases. Among the 61 *BRCA1*-positive patients, the majority of tumors were TNBC (61%, 37/61). HER2-positive carcinomas were identified in 15% (9/61), and HR+/HER2− carcinomas in 25% (15/61). In the 53 *BRCA2*-positive patients, nearly half (26/53) had HR+/HER2− tumors, 28% had TNBC (15/53), and 23% (12/53) had HER2-positive carcinomas. Overall, the distribution of tumor subtypes differed significantly between genetic subgroups, with TNBC being significantly enriched among *BRCA1* mutation carriers, whereas *BRCA2*-positive and mutation-negative patients more frequently presented with HR+/HER2− tumors (chi-square test, *p* = 0.017). In patients with negative genetic test results (*n* = 184), HR+/HER2− carcinomas were also the most common subtype, accounting for 54% (100/184) of cases. TNBC was diagnosed in 22% (41/184) and HER2-positive tumors in 23% (43/184). These findings highlight that TNBC was more prevalent in the *BRCA1*-positive cohort, whereas *BRCA2*-positive patients more frequently presented with HR+/HER2− carcinomas. Notably, the tumor subtype distribution in *BRCA2*-positive patients closely resembled that of patients with negative genetic test results. Figure 3 illustrates the distribution of tumor subtypes across *BRCA1*-positive, *BRCA2*-positive, and genetically negative patient groups.

**Fig. 3.**
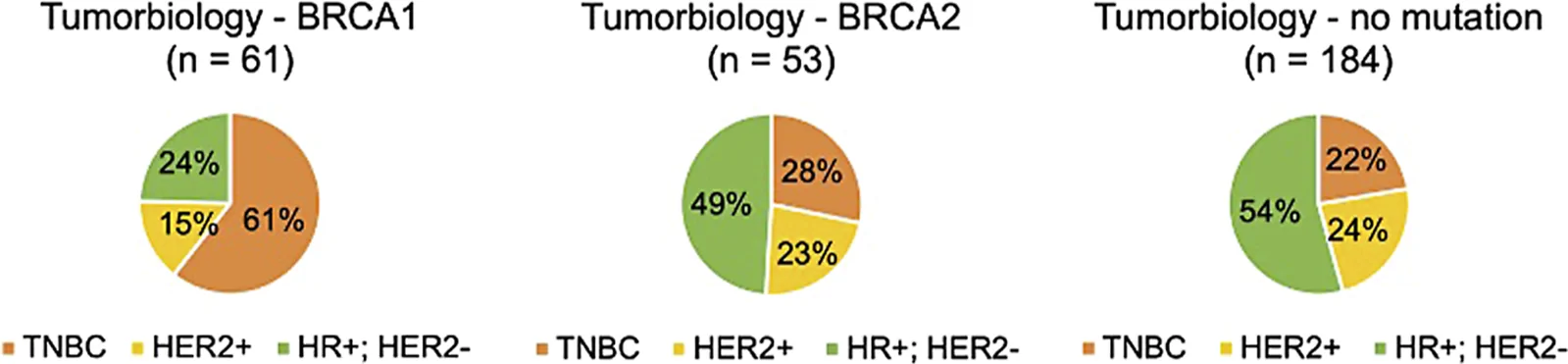
Distribution of tumor subtypes in the BRCA1, BRCA2, and genetically negative groups.

### Chemotherapy

Table 2 summarizes the use of CT across cohorts stratified by genetic testing and tumor biology. Overall, the majority of patients (76%, 257/338) received systemic CT. Of these, 56% (144/257) were treated neoadjuvantly and 41% (105/257) adjuvantly. In the *BRCA1*-positive cohort, 89% (54/61) received CT. Within this group, neoadjuvant CT (NACT) was administered slightly more frequently (54%, 29/54) than adjuvant CT (41%, 22/54), while treatment data were missing in 6% of cases (3/54). Notably, all *BRCA1*-positive patients with TNBC or HER2-positive tumors received CT. Among those with hormone receptor-positive, HER2-negative tumors, 92% (12/13) underwent CT (data not shown). In *BRCA2*-positive patients, 75% (40/53) received CT. Neoadjuvant and adjuvant therapies were administered nearly equally: 50% (20/40) and 48% (19/40), respectively. All *BRCA2*-positive patients with TNBC received CT, while 82% of those with HER2-positive tumors and 85% of those with hormone receptor-positive, HER2-negative tumors, were treated with CT. Among patients with negative genetic testing, 73% (134/184) received CT. Of these, 57% (77/134) received neoadjuvant therapy, 40% (54/134) adjuvant therapy, and 2% (3/134) had missing treatment data. CT was predominantly administered in the TNBC (97%, 36/37) and HER2-positive (92%, 36/39) subgroups, while 75% (62/83) of patients with hormone receptor-positive, HER2-negative tumors underwent CT. The use of platinum-based CT evolved over time, with a marked increase observed in the TNBC group since 2014 (Fig. 4). Among TNBC patients, the proportion receiving platinum-containing regimens steadily rose, reaching 100% (14 out of 14) by 2020.

**Table 2.** Use of CT according to genetic subgroup and tumor subtype

A. Genetic subgroups					
Group	CT	No CT	NACT	Adjuvant	Platinum-based CT
*BRCA1* (*n* = 61)	54 (89%)	1 (2%)	29 (54%)	22 (41%)	19 (35%)
*BRCA2* (*n* = 53)	40 (75%)	5 (9%)	20 (50%)	19 (48%)	9 (23%)
No mutation (*n* = 184)	134 (73%)	25 (14%)	77 (57%)	54 (40%)	28 (21%)

B. Tumor biology subgroups					
Group	CT	No CT	NACT	Adjuvant	Platinum-based CT
TNBC (*n* = 100)	91 (91%)	1 (1%)	62 (68%)	26 (29%)	48 (56%)
HER2+(*n* = 78)	65 (80%)	7 (9%)	46 (71%)	26 (17%)	10 (16%)
HR+/HER2−(*n* = 160)	101 (63%)	32 (20%)	36 (36%)	62 (61%)	6 (6%)

Percentages refer to the respective subgroup total. Cases with missing treatment information are not shown in the table.

Percentages refer to the respective subgroup total. Cases with missing treatment information are not shown in the table.

**Fig. 4.**
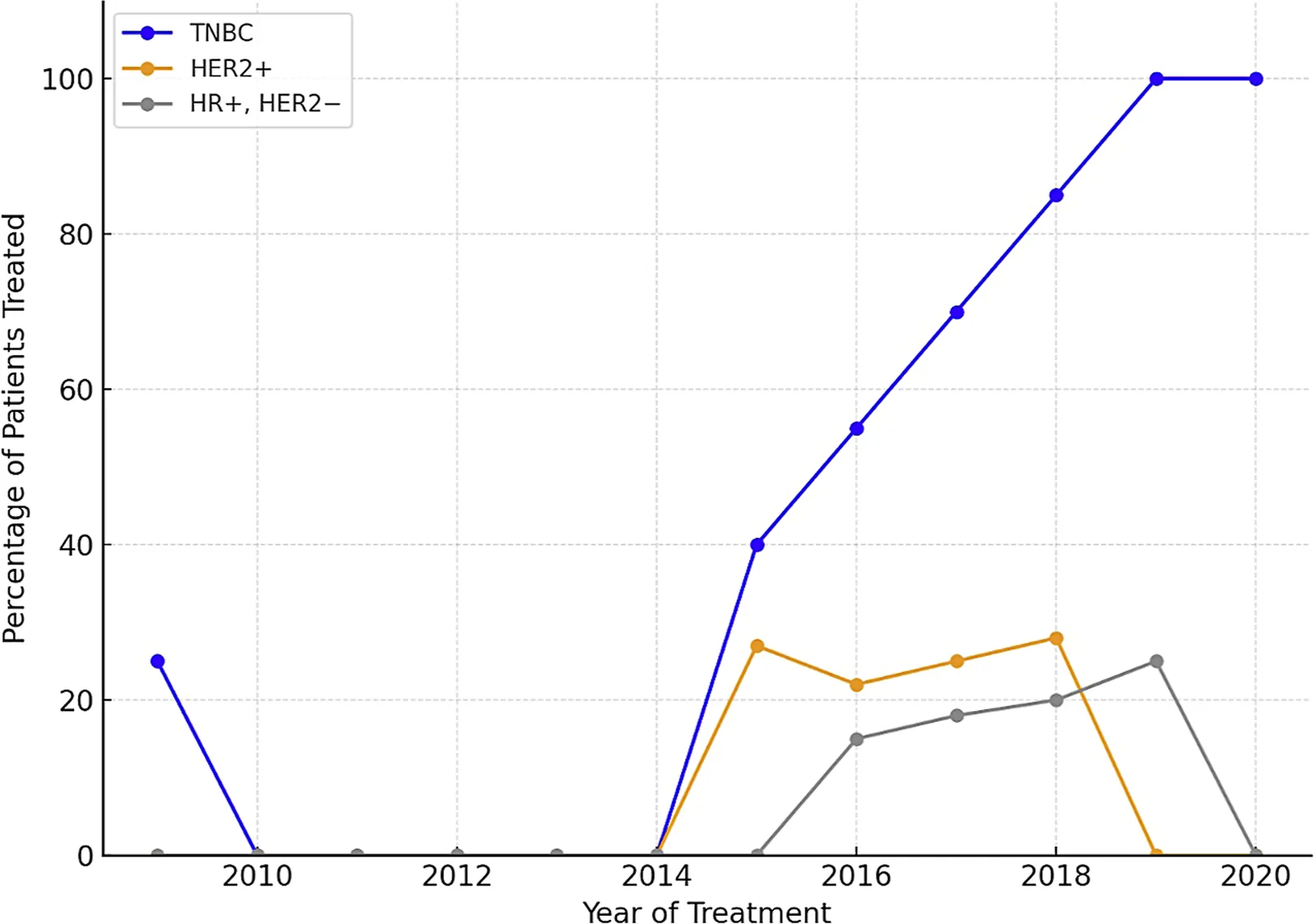
Use of platinum-based CT in the study cohort.

### pCR Rates

A total of 144 patients received NACT, of whom 22% (31/144) achieved a pCR, while 31% (45/144) did not. For approximately half of the patients (68/144), pCR status could not be determined due to missing data. This high proportion of missing data is attributable to the timing and setting of data collection: patient information was recorded at the time of counseling or genetic testing at our center, whereas breast cancer treatment was often carried out externally at cooperating hospitals. Consequently, only clinical data available at the time of presentation were accessible, and in many cases, NACT had not yet been completed, precluding assessment of pCR.

Among patients receiving NACT and with available response data, patients with pathogenic *BRCA1* variants demonstrated the highest pCR rate (74%, 14/19), compared with *BRCA2*-positive patients (50%, 4/8) and mutation-negative patients (33%, 14/42). The difference was not statistically significant (Fisher’s exact test, *p* = 0.347), likely due to the limited sample size. When stratified by tumor subtype, pCR was observed most frequently in TNBC (53%, 19/36), followed by HER2-positive tumors (43%, 10/23) and HR+/HER2− tumors (12%, 2/17). Differences between tumor subtypes were statistically significant (Fisher’s exact test, *p* = 0.007).

## Discussion

This retrospective cohort study examined the spectrum of germline mutations, systemic therapy patterns, tumor biology distribution, and response to NACT in 385 patients with an elevated familial risk for breast cancer treated at the Regensburg Center of the GC-HBOC between 2012 and 2020. A genetic cause was identified in approximately half of the patients, while 50.9% tested negative. Pathogenic *BRCA1/2* mutations were detected in 36% of cases, reflecting the effective implementation of risk stratification protocols according to GC-HBOC guidelines [2]. *BRCA1* mutations were slightly more frequent than *BRCA2* mutations, consistent with prior reports [9]. Additionally, 13% of patients carried pathogenic variants in other moderate-risk *BRCA* genes such as *CHEK2*, *PALB2*, and *ATM* [4]. These findings underscore the genetic heterogeneity of hereditary breast cancer and emphasize the clinical relevance of multigene panel testing beyond *BRCA1/2* [10]. The mutation rate observed in our cohort appears comparatively high when contrasted with unselected breast cancer populations, in which *BRCA* mutation frequencies are typically reported at 5–10% [11, 12]. However, this finding can be explained by the specific characteristics of our study population. Only patients fulfilling the inclusion criteria of the GC-HBOC were included, resulting in a cohort enriched for individuals with a high familial risk. In addition, as a tertiary referral center, our institution likely receives a higher proportion of patients from high-risk families, further contributing to an increased mutation detection rate. Similar variability in *BRCA* mutation frequencies has been reported in other studies focusing on high-risk populations, with rates ranging from approximately 16% to over 40%, depending on selection criteria and family history constellations [13–15]. These findings are consistent with reports from GC-HBOC-associated centers, where mutation detection rates are substantially higher than in unselected cohorts due to strict application of inclusion criteria and targeted genetic testing strategies [4, 13]. Taken together, our results are in line with the existing literature and reflect the expected mutation spectrum in a highly selected high-risk population. Tumor subtype distribution varied significantly across genetic subgroups, consistent with established genotype-phenotype correlations. Pathogenic *BRCA1* variants carriers most frequently presented with TNBC, whereas *BRCA2*-positive patients predominantly exhibited HR+/HER2− tumors, a pattern closely resembling that of mutation-negative cases. Interestingly, the proportion of HER2-positive tumors in our cohort was somewhat higher than generally reported in the literature, particularly among BRCA1 mutation carriers. While BRCA1-associated breast cancers are classically characterized by a predominantly triple-negative phenotype and infrequent HER2 overexpression, 15% of BRCA1-positive and 23% of BRCA2-positive patients in our cohort presented with HER2-positive disease. Several factors may account for this observation, including the retrospective real-world design, the relatively small subgroup sizes, and the inclusion of all patients fulfilling GC-HBOC testing criteria rather than selected biological subgroups [4, 6]. Overall, 76% of patients (76%) received CT, with the highest proportion observed among carriers of *BRCA1* pathogenic variants (89%). Nearly all patients with TNBC or HER2-positive tumors underwent CT, irrespective of mutation status, reflecting guideline-concordant management [16]. Notably, in the HR+/HER2− subgroup with pathogenic *BRCA* variants, CT was also frequently administered, a finding of clinical relevance, as a neoadjuvant approach enables subsequent consideration of post-neoadjuvant PARP inhibitor therapy [5, 17]. The proportion of patients receiving NACT increased over time, particularly among those with TNBC, where 68% were treated neoadjuvantly. This trend reflects growing recognition of the prognostic significance of pCR and the potential for treatment escalation or de-escalation based on response in high-risk disease. Our findings align with shifts in clinical practice following pivotal trials such as GeparSixto, which demonstrated improved pCR rates and event-free survival with the addition of carboplatin in TNBC [18]. By 2020, all TNBC patients at our center received platinum-containing regimens, underscoring the rapid integration of trial evidence into routine practice. The therapeutic benefit of platinum compounds is mechanistically linked to homologous recombination repair deficiencies, particularly in *BRCA1/2-*mutated tumors, explaining the marked chemosensitivity observed in these cancers [19, 20]. Among patients who received NACT and had available response data, the highest pCR rate was observed in carriers of pathogenic *BRCA1* variants (74%), compared with 50% in *BRCA2*-positive patients and 33% in patients without detectable genetic alterations. Within the *BRCA1*-positive cohort, 67% of patients with *BRCA1*-mutated TNBC achieved pCR. Similarly, *BRCA2*-mutated TNBC patients demonstrated high response rates, with pCR observed in 60% of cases, although the number of patients was limited. Favorable responses were also noted in HER2-positive patients with *BRCA* mutations; however, case numbers were small. In contrast, no increase in pCR rates was observed in *BRCA*-associated HR+/HER2− tumors compared with sporadic cases, indicating that CT sensitivity in this subtype may not be substantially influenced by germline mutation status [21]. Consequently, CT decisions for HR+/HER2− cancers should continue to be guided by established clinicopathologic criteria rather than germline status alone. These findings are consistent with previously published evidence on the efficacy of CT in *BRCA*-associated breast cancer. Multiple studies and meta-analyses have demonstrated higher pCR rates among *BRCA* mutation carriers compared with noncarriers following NACT. In a recent systematic review of 13 neoadjuvant studies, Jia et al. [22] reported a 17.6% higher likelihood of achieving pCR in patients with pathogenic *BRCA1/2* variants compared with wild-type counterparts.

A large propensity score-matched cohort study by Kim et al. [23] further substantiated these observations, reporting a pCR rate of 40% in *BRCA1/2* carriers compared with 26.5% in noncarriers, accompanied by improved distant metastasis-free survival. In contrast, other analyses indicate that this increased chemosensitivity is primarily confined to *BRCA1*-associated tumors, whereas *BRCA2*-mutated cancers often exhibit pCR rates comparable to sporadic cases, reflecting inherent differences in tumor biology [21]. Overall, our real-world findings align closely with existing evidence, particularly regarding the enhanced chemosensitivity of *BRCA1*-associated tumors and TNBC. These results have several important clinical implications. First, CT indication should not be based solely on germline mutation status; systemic therapy decisions must continue to follow established standard-of-care criteria. Second, when CT is indicated, the neoadjuvant approach should be preferred, not only for its prognostic and surgical advantages but also because it allows time for genetic testing and enables post-neoadjuvant strategies, such as PARP inhibitor therapy, as demonstrated in the OlympiA trial [5]. Third, while platinum-based CT is now well established in TNBC, its role in HR+/HER2− *BRCA*-associated tumors remains uncertain. Translational data suggest that homologous recombination deficiency may also confer platinum sensitivity in a subset of HR+ cancers [19, 24]; however, robust randomized evidence is lacking. Thus, this concept should currently be regarded as hypothesis-generating and merits further prospective evaluation. The novelty of our study lies in the systematic, real-world evaluation of a large, genetically characterized high-risk cohort. Our data underscore that germline testing is beyond prevention and risk-reduction strategies, and essential for post-neoadjuvant treatment decisions; however, the presence of pathogenic variants alone does not warrant modification of primary systemic therapy. At the same time, our data generate clinically relevant hypotheses, particularly regarding the possible role of platinum-based CT in HR+ *BRCA*-associated cancers. Despite these clinically relevant findings, several limitations have to be considered when interpreting the results. A limitation of this study is the incomplete availability of pCR data. Approximately half of the patients undergoing NACT lacked documented pCR status. This is primarily explained by the structure of the study cohort, which includes patients referred from external institutions for genetic counseling and testing. In these cases, patients were often evaluated before completion of systemic therapy, and follow-up data regarding treatment response were therefore not available at our center. Due to data protection regulations, these data could not be retrospectively retrieved from external institutions. Consequently, analyses of pCR should be interpreted with caution and considered exploratory and hypothesis-generating rather than confirmatory. In addition, the evolution of genetic testing strategies over the study period should be considered when interpreting mutation frequencies. The transition from *BRCA1/2*-only testing to comprehensive multigene panel diagnostics may have contributed to an increased detection of pathogenic variants in later years and thus may have influenced the observed mutation rates. Patients who had undergone genetic testing prior to 2015 were invited to participate in expanded multigene panel testing through a structured recall system. However, uptake of retesting was not systematically assessed in this study and was likely incomplete. This may have contributed to an underestimation of mutation prevalence in the early study period. Changes in GC-HBOC inclusion criteria over time were minor and are unlikely to have substantially influenced mutation detection rates in this cohort. However, as patients were not systematically re-invited following later expansion of the criteria, a potential bias in mutation prevalence estimates cannot be excluded. Finally, this was a retrospective single-center study, which may limit the generalizability of the findings. The study population represents a highly selected high-risk cohort treated at a tertiary referral center, which may further contribute to differences compared with population-based cohorts. However, the study reflects real-world clinical practice.

In conclusion, our results indicate that systemic therapy in high-risk patients should continue to follow standard treatment principles applied to sporadic breast cancers. We demonstrated that routine care in an GC-HBOC center facilitates the rapid implementation of emerging evidence, such as the adoption of platinum-containing regimens in TNBC. While germline mutations are highly informative for screening and post-neoadjuvant treatment planning, they should not influence initial CT decisions. Future clinical trials are warranted to determine whether platinum-based therapy can enhance chemosensitivity in HR+ *BRCA*-associated tumors and expand therapeutic options in this patient subgroup.

## Statement of Ethics

This study was carried out in accordance with The Code of Ethics of the World Medical Association (Declaration of Helsinki). All members of the research team committed themselves to the confidentiality of the information provided as well as to data protection and are subject to medical confidentiality. The study was reviewed and approved by the Ethics Committee of the University of Regensburg, Approval No. 21-2297-104. Written informed consent from participants was not required in accordance with local guidelines as this was a retrospective cohort study.

## Conflict of Interest Statement

Maria Hatzipanagiotou received speaking and lecture fees from Novartis, Lilly, Roche, Pfizer, RG Ärztefortbildung and AstraZeneca within the past 3 years. Lisa Witterauf, Mikosch Augele, Aviva Fischer, Ines Schönbuchner, Simone Hammer, Miriam Fernandez-Pacheco, Deborah Huber, Bernhard H.F. Weber, and Davor Lessel have no competing interests to declare. Ivana Holzhauser has received lecture fees from AstraZeneca. Stephan Seitz has served as a consultant for AstraZeneca, Daiichi Sankyo, Gilead, GSK, Lilly, MSD, Novartis, Pfizer, Roche, and Menarini Stemline; received honoraria for speaking engagements from AstraZeneca, Daiichi Sankyo, GE Healthcare, Gilead, GSK, Lilly, MSD, Novartis, Pfizer, Roche, and Menarini Stemline; as well as received support for congress attendance and/or travel from AstraZeneca, GE Healthcare, Gilead, Lilly, Pfizer, and Menarini Stemline within the past 3 years. Olaf Ortmann has received honoraria and reimbursement of travel expenses from iOMEDICO AG, Jörg Eickeler, Forum für medizinische Fortbildung (FomF GmbH), Astellas Pharma, Novartis Pharma, RG Ärztefortbildung, Med Update GmbH, Aurikamed, AstraZeneca, LAWG Deutschland e.V., and Eisai.

## Funding Sources

The authors declare that no funds, grants, or other support were received during the preparation of this manuscript.

## Author Contributions

Maria Eleni Hatzipanagiotou: conceptualization, supervision, and writing – original draft. Lisa Witterauf: protocol development, software, data curation, formal analysis, and visualization. Mikosch Augele: data curation, formal analysis, and visualization. Aviva Fischer: data curation and management. Ivana Holzhauser: data curation and supervision. Ines Schönbuchner, Simone Hammer, and Deborah Huber: conceptualization and supervision. Miriam Fernandez-Pacheco: writing – review and editing. Bernhard Weber: methodology and supervision. Davor Lessel: validation and supervision. Olaf Ortmann: writing – review and editing, and supervision. Stephan Seitz: conceptualization, supervision, project administration, writing – review and editing, data management, and formal analysis.

## Data Availability

The data that support the findings of this study are available on request from the corresponding author. The data are not publicly available due to privacy or ethical restrictions.
